# A study of COVID-19 vaccination in the US and Asia: The role of media, personal experiences, and risk perceptions

**DOI:** 10.1371/journal.pgph.0000734

**Published:** 2022-07-13

**Authors:** Kaitlyn B. Akel, Grace A. Noppert, Yogambigai Rajamoorthy, Yihan Lu, Awnish Singh, Harapan Harapan, Hao-Yuan Chang, Felicia Zhang, Shu-Fang Shih, Abram L. Wagner

**Affiliations:** 1 Department of Epidemiology, School of Public Health, University of Michigan, Ann Arbor, MI, United States of America; 2 Institute for Social Research, University of Michigan, St. Ann Arbor, MI, United States of America; 3 Faculty of Accountancy and Management, Department of Economics, Universiti Tunku Abdul Rahman, Selangor, Malaysia; 4 Ministry of Education Key Laboratory of Public Health Safety, Department of Epidemiology, Fudan University School of Public Health, Shanghai, China; 5 National Technical Advisory Group on Immunisation Secretariat, National Institute of Health and Family Welfare, New Delhi, India; 6 Medical Research Unit, School of Medicine, Universitas Syiah Kuala, Banda Aceh, Indonesia; 7 Department of Microbiology, School of Medicine, Universitas Syiah Kuala, Banda Aceh, Indonesia; 8 Tropical Disease Centre, Universitas Syiah Kuala, Banda Aceh, Indonesia; 9 Tsunami & Disaster Mitigation Research Center (TDMRC), Universitas Syiah Kuala, Banda Aceh, Indonesia; 10 School of Nursing, College of Medicine, National Taiwan University, Taipei, Taiwan; 11 Department of Nursing, National Taiwan University Hospital, Taipei, Taiwan; 12 Department of Health Administration, College of Health Professions, Virginia Commonwealth University, Richmond, VA, United States of America; CSIR-Indian Institute of Chemcial Technology, INDIA

## Abstract

The COVID-19 pandemic presents an opportunity to assess the relationship between personal experiences and vaccine decision-making. The aim of this study was to examine the associations between experiences with COVID-19 and COVID-19 vaccination status. We administered 28 repeated cross-sectional, online surveys between June 2020 and June 2021 in the US and Asia. The main exposure was media showing COVID-19 cases, and we distinguished those with no such experience, those seeing a not severe case of disease, and those seeing a severe case of disease. Logistic regression models estimated the association between experience and acceptance of a hypothetical COVID-19 vaccine (pre-rollout) or actual vaccination (post-rollout). We explored perceived susceptibility as a potential mediator. Intent to vaccinate was lowest in the US and Taiwan, and highest in India, Indonesia, and China. Across all countries, seeing a severe case of COVID-19 in the media was associated with 1.72 times higher odds of vaccination intent in 2020 (95% CI: 1.46, 2.02) and 2.13 times higher odds of vaccination in 2021 (95% CI: 1.70, 2.67), compared to those not seeing a case or a less severe case. Perceived susceptibility was estimated to mediate 25% of the relationship with hypothetical vaccination (95% CI: 18%, 31%, P<0.0001), and 16% of the relationship with actual vaccination 16% (95% CI: 12%, 19%, P<0.0001). Seriousness of experiences could relate to intention to vaccinate against COVID-19. Media exposures are a modifiable experience, and this study highlights how this experience can relate to risk perceptions and eventual vaccination, across a variety of countries where the course of the pandemic differed.

## Introduction

The coronavirus disease 2019 (COVID-19) pandemic, caused by severe acute respiratory syndrome coronavirus 2 (SARS-CoV-2) has resulted in a number of social and economic upheavals [1,2]. Globally, vaccination rates vary greatly across countries. As of May 21, 2021, 10% of the world was vaccinated, with wide ranges across countries. For example, <6% of Indonesia, Malaysia, or Taiwan was vaccinated, compared to over 50% for the US. However, by May 2022, a larger proportion in Asia (75%) had been vaccinated than in North America (72%) [3].

Although there have been some cross-national studies of parents’ hesitancy towards pediatric vaccines [4–6], less is known about hesitancy and acceptance of adult vaccines worldwide. Studies during the 2009 H1N1 pandemic found vaccine acceptance varied, and ultimately waned over the course of the pandemic [7,8]. Vaccine acceptance could correlate with risk perceptions [9], and during the H1N1 pandemic perceived risk varied with actual case counts, but trended downward [8], likely as individuals became more familiar with the disease.

Lived experiences with the pandemic (e.g., contracting infection oneself, or knowing someone with a severe case of disease) could interact with risk perceptions. In this way, analytical and experiential systems for processing risk could operate in parallel [10]. Overall, research on the connections between experiences with SARS-CoV-2 infections and vaccine acceptance is limited, even though experiences have been hypothesized to be a key part of vaccine decision-making [9].

Media experiences could be an important modifiable exposure. What individuals see or do not see in the media could impact their own risk perceptions and ultimately their own behaviors. For example, early studies of media found that individuals who watched more crime news perceived themselves to be at higher risk for being a crime victim [11]. Previous research has focused on how the breadth of exposure could impact risk perceptions [12], or how media exposure varies in effect by demographic group [13]. For COVID-19, much of the current media environment has focused on politicians and statistics [14]. However, it could be that social or traditional media that highlights the severity of COVID-19 illness could be more effective in promoting vaccination.

Given the need to better understand how individuals make decisions about getting vaccinated, the purpose of this study was to assess the role of individual experiences with COVID-19 on vaccine intent and uptake in the US and Asia. We examined the mediating role of perceived susceptibility to viral infection and mortality in this pathway. Experiences with COVID-19 were measured across three dimensions: whether an individual was diagnosed with COVID-19, whether they knew family or friends who were, or whether they had seen COVID-19 cases in social or traditional media.

## Methods

### Ethical approval

The protocol was reviewed and approved by ethical review committees in each country, including the University of Michigan Health Sciences and Behavioral Sciences Institutional Review Board (#HUM00180096), the Fudan University School of Public Health ethical review committee (#IRB00002408), the National Taiwan University Hospital Research Ethics Committee (#202007102RINB), the Universiti Tunku Abdul Rahman (#U/SERC/107/2020), the Komite Etik Penelitian Kesehatan at Universitas Syiah Kuala (#041/EA/FK-RSUDZA/2020), and the Sigma-IRB in New Delhi, India (#10003/IRB/20-21). Participants read an informed consent form and clicked “I agree to participate in the study” prior to any data collection occurring.

### Study population

We recruited participants through Dynata, an independent survey research firm and used convenient, opt-in sampling [15]. We also instated age and gender quotas during recruitment, ensuring that the number of participants invited to take the online survey approximately matched the age and gender distribution of each country’s adult population. The survey itself was advertised through social media and was entirely internet-based. Participants over 18 years old were considered eligible in all countries, except Taiwan, where those 20 years old and older were eligible, because at the time the study was reviewed by an ethical review committee, the age of majority was 20. We used Qualtrics as the platform to collect data from the participants in all six countries. We attempted to obtain a sample size of 800 at each wave. With an alpha of 0.05 and a power of 80%, a sample size of 800 could estimate an outcome proportion of 50%, a statistically conservative estimate of the population vaccinated, at a sufficiently precise interval, with a margin of error of 4%.

We collected data from six countries including the US, China, Taiwan, Malaysia, Indonesia, and India in August and November in 2020, and March and June 2021. In June and October 2020, and February and April 2021, we also collected additional waves in the United States. For data quality control, we deleted data for those who did not consent, did not complete up to the last section of the survey, or took less than 3 minutes to complete the questionnaire. All questionnaires are available at: https://doi.org/10.6084/m9.figshare.14792058.

The six sites of data collection (US, China, Taiwan, Malaysia, Indonesia, and India), all have distinct socioeconomic and demographic contexts and unique trajectories with COVID-19, and together represent 44.6% of the world’s population [16]. Each country also has specific characteristics, including different governmental responses [17] and COVID-19 epicurves [18], that make it an important place to study the experiences of COVID-19 and vaccine hesitancy.

### Main exposure

The primary independent variable was the severity of experiences with COVID-19 infections, ranging from mild to very serious. We asked participants about their experiences during the pandemic, such as whether or not they had an infection, or witnessed a close friend or relative with COVID-19, or witnessed someone in the media contracted COVID-19. Then participants were asked about the seriousness of these cases from their perspective (e.g., “How serious was the course of illness when you were infected with the novel coronavirus?”). We then generated three separate, independent, three-level variables corresponding to individuals’ reported experiences:

- **Participants personal experience with COVID-19:** Never personally diagnosed with COVID-19; Diagnosed with COVID-19 but was not a severe case; Diagnosed with COVID-19 and was a very severe case.
- **Participants’ experience with COVID**-**19 in their network of family/friends:** No experience with someone in their network diagnosed with COVID-19; Someone in their network was diagnosed with COVID-19 but was not a severe case; Someone in their network was diagnosed and was a very severe case.
- **Participants’ experience with COVID-19 through the media:** No media exposure to someone being diagnosed with COVID-19; Media exposure to a case of COVID-19 but not a severe case; Media exposure to a severe case of COVID-19.

For our formal mediation analysis, we dichotomized experiences with COVID-19 to improve interpretability of the results. Due to limited numbers of individuals diagnosed with COVID-19 in some countries, we did not separate out severity of illness for an individual’s personal diagnosis.

For aid of interpretation of mediation analyses, we collapsed media experiences into two levels: seeing a severe case in media vs seeing a non-severe case or not seeing any case.

### Mediator

The mediator assessed in this study was individuals’ own risk perceptions regarding COVID-19, specifically their perception regarding their susceptibility to COVID-19 and their perceived risk of dying of COVID-19. In the survey, perceived susceptibility was measured continuously, by asking participants what they thought was the likelihood they would contract COVID-19, followed by the likelihood they would die from COVID-19. Both were measured using a sliding scale from 0% to 100%.

### Control variables

Income was dichotomized at an amount roughly equivalent to $2,000/month at purchasing power parity (7,500 RMB in China, 30,000 New Taiwan Dollars in Taiwan, 40,000 rupees in India, and 3,000 ringgits in Malaysia). We also adjusted for urbanicity (urban vs rural, based on respondent self-report), and age (trichotomized to 18–34, 35–54, and ≥55 years).

As risk perceptions could be influenced by behaviors already undertaken [19], we additionally adjusted for two categories of behaviors. We asked respondents how many days in the past week they went to school/work outside the home, or to the grocery store / another food vendor. If they responded ≥1 day, they were asked if they wore a mask all the time, some of the time, or never. For both sets of variables, we categorized respondents into 3 groups: those who did not go out, those who went out and always wore a mask, and those who went out but sometimes/never wore a mask.

### Outcome

For the datasets from 2020, the outcome was a binary indicator representing a participant’s intention towards COVID-19 vaccination. We measured the intention by asking participants if they would accept a vaccine under a randomized set of safety and efficacy levels [20].

In 2021, we instead asked individuals if they had been vaccinated. For those who had not been vaccinated, we subsequently asked, “How much do you agree or disagree with this statement? I plan to get a COVID-19 vaccine.” For our outcome, we combined those already vaccinated and those agreeing or strongly agreeing in a plan to get the vaccinate, as a measure comparing those already vaccinated or planning to vaccinate vs those with no such plans.

### Statistical analyses

Our analysis was designed in stages to do the following: (1) to test the relationship between the exposure (COVID-19 experiences) and a dichotomous outcome (vaccination), (2) to test the relationship between COVID-19 experiences and the mediator (perceived susceptibility to SARS-CoV-2 infection), and (3) to test significance of a full model including mediator as a covariate with vaccination as the outcome. The first two sets of models were conducted separately across country, and the third, full, model used all countries’ data together.

Using the data from 2020, we first estimated the direct effect of the exposure variables (experiences with COVID-19) on the primary outcome of intention to vaccinate against COVID-19. We used a series of logistic regression models to produce odds ratios (ORs) and 95% confidence intervals (CI) separately for each country. The models controlled for wave of data collection, gender, income, age, work/school behaviors, grocery store behaviors, and vaccine safety and effectiveness profiles.

To understand what factors could potentially mediate this association, we estimated a series of models examining the relationship between the exposure (experiences with COVID-19), and one particularly salient potential mediator, individuals’ perceived susceptibility to COVID-19. To do so, we constructed a series of linear regression models estimating the marginal mean risk perception of both susceptibility to SARS-CoV-2 and risk of dying due to COVID-19 associated with each of the exposure variables. These models were also separated by country. These models controlled for wave of data collection, gender, urbanicity, income, age, work/school behaviors, and grocery store behaviors.

Our final set of models collapsed together results from all six study sites. A mediation model was constructed with the predictor of media experiences (i.e., the experience in our study which could be the target of interventions like health promotions). We present results of two sets of two logistic regression models. The first set used waves from 2020, with the outcome of vaccination intent. Within this set, one model adjusted for confounders/control variables only, and the second adjusted also for potential mediation from perceived susceptibility. The second set of models used waves of 2021, and the outcome of already or planning to vaccinate. We estimate the proportion mediated through the CAUSALMED procedure in SAS [21].

We created population weights for age, sex, and region using the raking macro in SAS [22]. Analyses were stratified by country, and all used appropriate survey procedures, including weights for the sample to be representative of the adult gender, age, and region of the country distribution and clusters based on wave. An alpha level of 0.05 was considered significant. Analyses were conducted in SAS version 9.4 (SAS Institute, Cary, NC, USA).

## Results

### Study population

Details about the sample size, the number who agreed to participate, and the number who finished the survey are available online (Table A [23]). Demographic characteristics of the study population are described in Table 1. In brief, the U.S. respondents were evenly divided across all eight waves of survey collection while each of the remaining five sites were evenly divided across four waves of survey collection. In total there were 22,870 participants included in the study, with over 630 in each wave of data collection.

**Table 1:** Demographic characteristics of study sample, stratified by country/region of sample.

	U.S.	Mainland China	Taiwan	Malaysia	Indonesia	India
Wave						
Jun 2020	657 (13%)	--	--	--	--	--
Aug 2020	783 (13%)	788 (25%)	645 (25%)	759 (25%)	727 (25%)	805 (25%)
Oct 2020	937 (12%)	--	--	--	--	--
Nov 2020	986 (13%)	939 (25%)	633 (25%)	738 (25%)	800 (25%)	957 (25%)
Feb 2021	877 (13%)	--	--	--	--	--
Mar 2021	917 (13%)	721 (25%)	679 (25%)	749 (25%)	789 (25%)	926 (25%)
Apr 2021	917 (13%)	--	--	--	--	--
Jun 2021	954 (13%)	971 (25%)	760 (25%)	779 (25%)	783 (25%)	894 (25%)
Gender: Male	3369 (50%)	1745 (51%)	1242 (48%)	1568 (52%)	1640 (51%)	1869 (52%)
Urbanicity: Rural	2264 (33%)	565 (18%)	353 (12%)	1312 (44%)	890 (27%)	629 (18%)
Income: Higher	5492 (77%)	3159 (91%)	2373 (90%)	2167 (70%)	1210 (38%)	1576 (43%)
Age						
18–34 years	1849 (34%)	1162 (34%)	1060 (29%)	1422 (43%)	1424 (41%)	1584 (43%)
35–54 years	2298 (35%)	1474 (40%)	1321 (43%)	1387 (41%)	1423 (41%)	1365 (38%)
≥55 years	2881 (30%)	783 (26%)	336 (28%)	216 (16%)	252 (18%)	633 (19%)
Work/school behavior in past week						
Did not go to work/school outside	3919 (51%)	404 (12%)	451 (18%)	829 (30%)	463 (14%)	853 (24%)
Went 1–3 days a week	1093 (18%)	406 (12%)	273 (10%)	649 (22%)	1052 (33%)	1310 (37%)
Went 4–7 days a week	2016 (31%)	2609 (76%)	1960 (72%)	1547 (49%)	1584 (53%)	1419 (39%)
Grocery store behavior in past week						
Did not go to grocery store	645 (9%)	104 (3%)	79 (3%)	223 (7%)	178 (6%)	266 (7%)
Went 1–3 days a week	5038 (70%)	2088 (61%)	1069 (40%)	1988 (66%)	1933 (63%)	2109 (59%)
Went 4–7 days a week	1345 (21%)	1227 (36%)	1547 (57%)	814 (27%)	988 (31%)	1207 (34%)
Diagnosed with COVID-19						
No	6019 (83%)	3339 (98%)	2645 (98%)	2823 (94%)	1837 (60%)	2302 (63%)
Yes, and was not severe	524 (8%)	57 (2%)	53 (2%)	81 (3%)	1130 (36%)	197 (6%)
Yes, and was very severe	485 (9%)	23 (1%)	19 (1%)	121 (4%)	132 (4%)	1083 (31%)
Friends/family diagnosed with COVID-19						
No	3916 (56%)	3266 (96%)	2655 (98%)	2193 (73%)	1730 (56%)	1071 (30%)
Yes, and was not severe	1932 (26%)	111 (3%)	43 (1%)	507 (17%)	1120 (36%)	1164 (32%)
Yes, and was very severe	1180 (17%)	42 (1%)	19 (1%)	325 (10%)	249 (7%)	1347 (39%)
Seen case of COVID-19 in media						
No	1460 (22%)	348 (10%)	591 (22%)	322 (11%)	123 (5%)	333 (9%)
Yes, and was not severe	2650 (37%)	2010 (59%)	1368 (50%)	1157 (40%)	1987 (64%)	1147 (31%)
Yes, and was very severe	2918 (42%)	1061 (30%)	758 (28%)	1546 (50%)	989 (32%)	2102 (60%)
Intending to receive a COVID-19 vaccine (Jun–Nov 2020)	2229 (68%)	1551 (90%)_	837 (70%)	1196 (79%)	1306 (86%)	1590 (90%)
Vaccination status (Feb–Jun 2021)						
Already vaccinated	1924 (49%)	1292 (75%)	128 (9%)	399 (26%)	678 (45%)	1180 (65%)
Plan to vaccinate	789 (23%)	330 (21%)	693 (48%)	879 (57%)	613 (38%)	491 (27%)
No plan to vaccinate	952 (27%)	70 (4%)	618 (43%)	250 (17%)	281 (17%)	149 (8%)

Among the U.S. survey respondents, 17% reported having been diagnosed with COVID-19 and 44% experienced a friend or family member with COVID-19. This is contrast to participants in mainland China, Taiwan, and Malaysia who reported lower experiences with COVID-19 both personally and among friends and family. However, 40% of survey respondents in Indonesia and 37% of respondents in India reported a diagnosis of COVID-19. Forty-four percent of respondents in Indonesia and 70% of respondents in India reported knowing a friend or family member diagnosed with COVID-19. Across all six country sites, a large proportion of respondents reporting seeing a case of COVID-19 in the media.

### Associations between experiences with COVID-19 and intention to vaccinate

**Fig 1:**
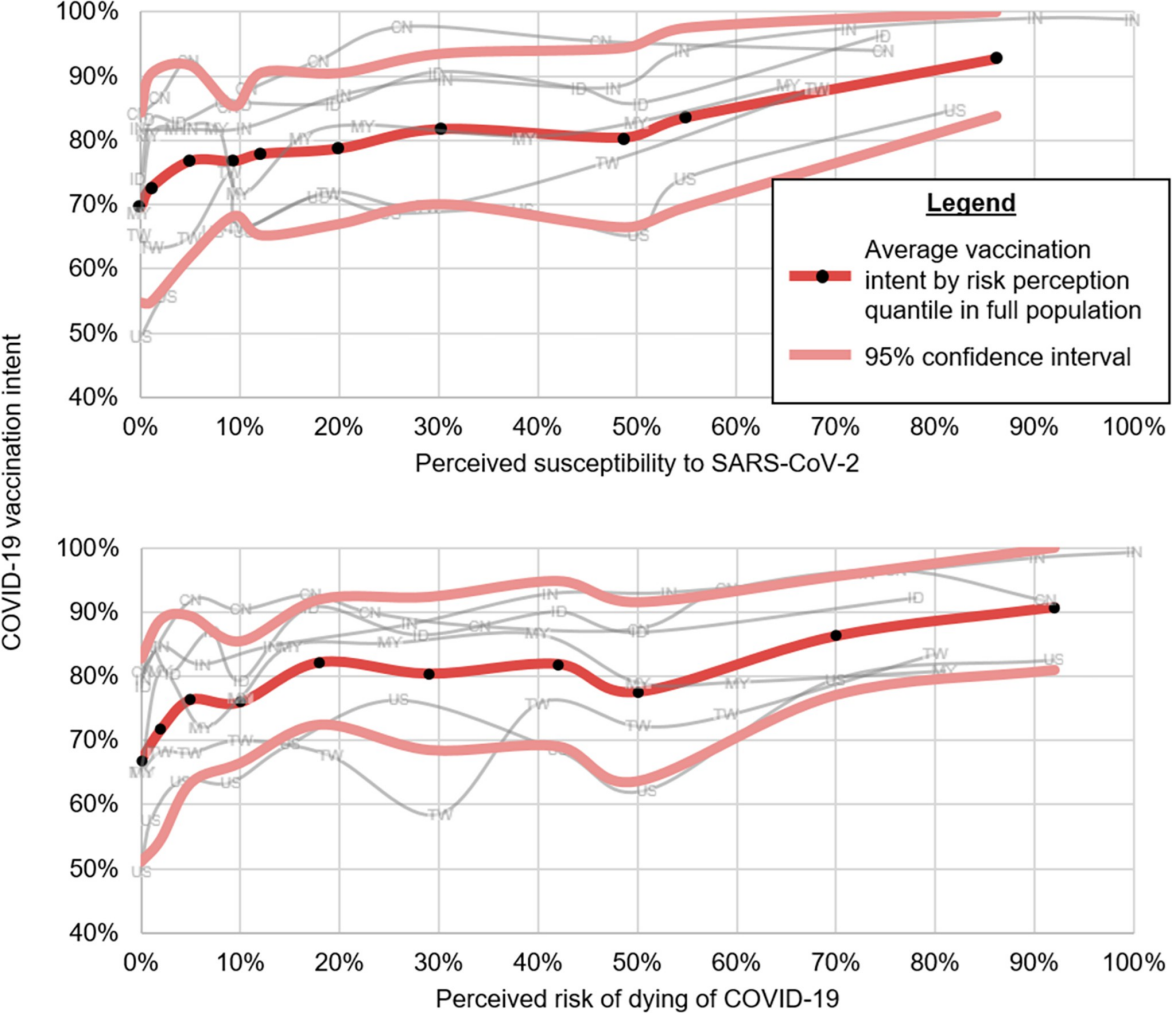
Relation between two measures of risk perception and intention to vaccinate by decile in the US, China, Taiwan, Malaysia, Indonesia, and India, June–November 2020 (N = 11,154).

Overall, intent to vaccinate was lowest in the US and Taiwan, and highest in India, Indonesia, and China, with Malaysia being in the middle. In general, we found higher risk perceptions were associated with higher vaccination intent. Fig 1 shows the average vaccination intent by decile in the total sample and within each country in 2020. For example, in the lowest decile of perceived susceptibility–which was 0% perceived susceptibility, 69% (95% CI: 54%, 84%) would accept a COVID-19 vaccine, but in the highest decile of perceived susceptibility (ranging from 86% to 99% susceptibility), 93% would accept a vaccine (95% CI: 84%, 100%). Similar patterns were found for perceived risk of dying. The lowest decile of perceived risk of dying (0%) had a vaccine acceptance of 67% (95% CI: 51%, 83%). In the highest decile (where perceived risk of dying ranged from 92% to 100%), vaccine acceptance was 91% (95% CI: 81%, 100%). For both figures there was a general monotonic relationship between increasing deciles of risk perception and greater vaccination intent, with some wobbliness at certain key percentages: e.g., there were dips around 10% and 50%. Some countries had other trends too, including dips for Taiwan around 30%, and decreased vaccination intent in Malaysia for those indicating their risk of dying was ≥50%.

Table 2 shows the associations between all three experiences with COVID-19 and vaccination intent using logistic regression models. By and large, reporting having been personally diagnosed with COVID-19 had the greatest impact on vaccination intent across country sites compared to knowing a friend or family member diagnosed with COVID-19 or media experiences. For example, in India participants that reported a personal diagnosis with COVID-19 had 12.95 times the odds (95% CI: 4.89, 34.28) of reporting they would accept a COVID-19 vaccine compared to those who were not personally diagnosed with COVID-19. In the US, having had a personal diagnosis of COVID-19 was associated with 1.84 times the odds (95% CI: 1.29, 2.62) of accepting a vaccine compared to those who were not personally diagnosed with COVID-19. S1 Table shows results from 2021 using the outcome of COVID-19 vaccination uptake, and showed similar patterns.

**Table 2:** Logistic regression models of intention to vaccinate by personal experiences, Jun–Nov 2020.

	U.S. OR (95% CI)	Mainland China OR (95% CI)	Taiwan OR (95% CI)	Malaysia OR (95% CI)	Indonesia OR (95% CI)	India OR (95% CI)
Sample size	3363	1727	1147	1496	1527	1762
Diagnosed with COVID-19						
No	1 (ref)	1 (ref)	1 (ref)	1 (ref)	1 (ref)	1 (ref)
Yes	1.84 (1.29, 2.62)	1.00 (0.24, 4.08)	2.69 (0.83, 8.74)	0.97 (0.38, 2.48)	1.27 (0.69, 2.37)	12.95 (4.89, 34.28)
Friends/family diagnosed with COVID-19						
No	1 (ref)	1 (ref)	1 (ref)	1 (ref)	1 (ref)	1 (ref)
Yes	1.16 (0.96, 1.41)	0.65 (0.21, 2.04)	0.61 (0.14, 2.58)	1.59 (0.96, 2.65)	1.22 (0.68, 2.20)	0.94 (0.62, 1.43)
Seen case of COVID-19 in the media						
No	1 (ref)	1 (ref)	1 (ref)	1 (ref)	1 (ref)	1 (ref)
Yes, and was not severe	0.97 (0.77, 1.22)	0.66 (0.36, 1.20)	1.07 (0.71, 1.60)	1.38 (0.84, 2.27)	1.97 (1.01, 3.84)	1.43 (0.78, 2.63)
Yes, and was very severe	1.67 (1.33, 2.11)	1.40 (0.73, 2.69)	1.57 (0.98, 2.50)	1.70 (1.04, 2.76)	3.11 (1.53, 6.31)	2.37 (1.26, 4.44)

Notes:

OR, odds ratio; CI, confidence interval.

Models adjusted for wave of data collection, gender, urbanicity, income, age, work/school behaviors, grocery store behaviors, and vaccine safety and effectiveness profile experiment.

Additionally, seeing a COVID-19 case in the media, particularly a severe case, was associated with increased odds of accepting a vaccine in the US, Taiwan, Malaysia, Indonesia, and India. The association was strongest among participants in Indonesia: those who experienced a severe case of COVID-19 in the media had 3.11 times the odds (95% CI: 1.53, 6.31) of accepting a vaccine compared to those who had not experienced a case of COVID-19 in the media. Knowing a friend or family member with COVID-19 was not associated with increased odds of vaccination acceptance across country sites.

### Associations between experiences with COVID-19 and perceived susceptibility to COVID-19 and COVID-19-related mortality

Marginal outcomes from linear regression models of risk perceptions are shown in Table 3. All three experiences were significantly associated with perceived susceptibility in the US. The mean perceived susceptibility to infection was lowest among those with no previous diagnosis (32%) and highest in those with a very severe case of COVID-19 (56%) (P = 0.0034). Similarly, knowing friends/family diagnosed with a severe case of COVID-19 led to the highest perceived susceptibility (50%), and was lower among those who knew a non-severe case (42%), or among those who did not know a case (38%) (P = 0.0019). In the US, seeing a non-severe case of COVID-19 in the media was associated with the lowest perceived risk of susceptibility to COVID-19 (40%) compared to not seeing a case of COVID-19 in the media (44%) or seeing a severe case of COVID-19 in the media (47%) (P = 0.0224).

**Table 3:** Marginal mean risk perceptions of COVID-19 (± standard error) by experiences in 6 countries/regions, June–November, 2020.

	U.S.	Mainland China	Taiwan	Malaysia	Indonesia	India
	Perceived susceptibility to SARS-CoV-2^a^					
Diagnosed with COVID-19	***P = 0*.*0034***	***P = 0*.*0252***	*P = 0*.*1913*	*P = 0*.*2042*	*P = 0*.*1783*	*P = 0*.*0731*
No	**32% ± 1%**	**24% ± 3%**	15% ± 1%	21% ± 0%	21% ± 3%	28% ± 3%
Yes, and was not severe	**42% ± 2%**	**49% ± 4%**	38% ± 6%	40% ± 6%	28% ± 1%	43% ± 5%
Yes, and was very severe	**56% ± 3%**	**35% ± 6%**	44% ± 26%	36% ± 8%	43% ± 2%	62% ± 1%
Friends/family diagnosed with COVID-19	***P = 0*.*0005***	*P = 0*.*3790*	*P = 0*.*6013*	*P = 0*.*0628*	*P = 0*.*3321*	*P = 0*.*7034*
No	**38% ± 1%**	29% ± 1%	35% ± 8%	30% ± 6%	32% ± 1%	42% ± 2%
Yes, and was not severe	**42% ± 2%**	34% ± 3%	42% ± 1%	34% ± 5%	31% ± 2%	43% ± 4%
Yes, and was very severe	**50% ± 1%**	45% ± 0%	20% ± 14%	33% ± 3%	29% ± 2%	48% ± 3%
Seen case of COVID-19 in media	***P = 0*.*0224***	*P = 0*.*1523*	*P = 0*.*9582*	*P = 0*.*1009*	*P = 0*.*9877*	***P = 0*.*0098***
No	**44% ± 2%**	40% ± 2%	31% ± 10%	32% ± 4%	29% ± 4%	**39% ± 3%**
Yes, and was not severe	**40% ± 1%**	32% ± 1%	30% ± 7%	33% ± 4%	29% ± 3%	**44% ± 3%**
Yes, and was very severe	**47% ± 1%**	36% ± 1%	36% ± 4%	32% ± 6%	34% ± 1%	**51% ± 4%**
	Perceived risk of dying of COVID-19^a^					
Diagnosed with COVID-19	***P = 0*.*0016***	*P = 0*.*1927*	***P = 0*.*0452***	*P = 0*.*0748*	*P = 0*.*0676*	*P = 0*.*0552*
No	**35% ± 1%**	39% ± 1%	**31% ± 9%**	30% ± 1%	25% ± 0%	25% ± 3%
Yes, and was not severe	**44% ± 4%**	59% ± 6%	**46% ± 10%**	45% ± 3%	29% ± 1%	43% ± 4%
Yes, and was very severe	**59% ± 2%**	40% ± 7%	**54% ± 15%**	44% ± 8%	38% ± 0%	65% ± 0%
Friends/family diagnosed with COVID-19	***P = 0*.*0020***	***P = 0*.*0124***	*P = 0*.*5162*	*P = 0*.*2559*	*P = 0*.*2790*	*P = 0*.*1174*
No	**42% ± 2%**	**42% ± 4%**	46% ± 11%	39% ± 3%	31% ± 1%	44% ± 3%
Yes, and was not severe	**42% ± 3%**	**48% ± 4%**	50% ± 6%	41% ± 4%	30% ± 2%	41% ± 3%
Yes, and was very severe	**54% ± 1%**	**49% ± 10%**	34% ± 20%	40% ± 5%	32% ± 2%	48% ± 1%
Seen case of COVID-19 in media	***P<0*.*0001***	*P = 0*.*4875*	*P = 0*.*0817*	*P = 0*.*3669*	*P = 0*.*4237*	*P = 0*.*3963*
No	**47% ± 2%**	41% ± 3%	38% ± 1%	39% ± 3%	33% ± 4%	43% ± 1%
Yes, and was not severe	**39% ± 2%**	45% ± 2%	41% ± 0%	37% ± 5%	26% ± 1%	41% ± 3%
Yes, and was very severe	**52% ± 1%**	53% ± 3%	52% ± 5%	43% ± 4%	33% ± 2%	49% ± 4%

Notes:

^a^ Linear regression models adjusted for wave of data collection, gender, urbanicity, income, age, work/school behaviors, and grocery store behaviors.

In the US, a similar pattern of results was observed examining perceived risk of dying of COVID-19. Experiences with COVID-19 (individual diagnosis, friends/family, and media) were associated with increased perceived risk of dying. Again, seeing a non-severe case of COVID-19 in the media was associated with the lowest perceived risk of dying of COVID-19 (39%) compared to not seeing a case in the media (47%) or seeing a severe case in the media (52%) (P<0.0001).

In mainland China, only personal experience with a COVID-19 diagnosis was associated with an increase in one’s perceived susceptibility to COVID-19 (P = 0.0252). However, having a friend or family member diagnosed with COVID-19 significantly impacted perceived risk of dying from COVID-19 in mainland China (P = 0.0124).

In Taiwan, those that reported having been diagnosed with COVID-19 had a greater perceived risk of dying from COVID-19 (46% for those with a non-severe case and 54% for those with a severe case) compared to those who were never diagnosed with COVID-19 (31%) (P = 0.0452).

For respondents in India, seeing a case of COVID-19 in the media was significantly associated with increased perceived susceptibility to COVID-19. Those that never experienced a case in the media had a 39% perceived susceptibility compared to seeing a non-severe case (44% perceived susceptibility) and those seeing a severe case (51% perceived susceptibility) (P = 0.0098).

For respondents in Malaysia and Indonesia, there were not significant associations between experiences with COVID-19 and perceived risk of susceptibility to or mortality related to COVID-19.

### Mediation of the experiences–vaccine intent relationship by risk perceptions

Table 4 shows models with and without perceived susceptibility, showing the direct and total estimated effect of media experiences on vaccination outcomes. Pre-vaccine rollout, each additional increase in perceived susceptibility was associated with 1.02 times higher odds of vaccination intent (95% CI: 1.02, 1.02). Media experiences were still significant, but slightly attenuated (OR: 1.56, 95% CI: 1.40, 1.75), corresponding to a proportion mediated of 25% (95% CI: 18%, 31%, P<0.0001). In 2021, post-vaccine rollout, there were similar directions of association, and the proportion mediated came to 16% (95% CI: 12%, 19%, P<0.0001).

**Table 4:** Estimates of total effect and direct effect of media experiences on COVID-19 vaccination intention in two logistic regression models.

	Intent to vaccinate (Aug and Nov 2020)		Already or plan to vaccinate (Mar and Jun 2021)	
	Adjusted model 1 (N = 9,428)	Adjusted model 2 (N = 9,380)	Adjusted model 1 (N = 9,853)	Adjusted model 2 (N = 9,774)
	OR (95% CI)	OR (95% CI)	OR (95% CI)	OR (95% CI)
Seen case of COVID-19 in media				
No / yes, and was not severe	1 (ref)	1 (ref)	1 (ref)	1 (ref)
Yes, and was very severe	1.72, (1.46, 2.02)	1.56, (1.40, 1.75)	2.13 (1.70, 2.67)	1.99 (1.58, 2.50)
Perceived susceptibility (continuous, %)	--	1.02, (1.02, 1.02)	--	1.01 (1.01, 1.02)
Wave: Nov 2020 vs Aug 2020	0.75, (0.67, 0.83)	0.73, (0.67, 0.81)	--	--
Wave: Jun 2021 vs Mar 2021	--	--	2.25 (1.47, 3.44)	2.16 (1.51, 3.09)
Gender: male vs female	1.08, (0.80, 1.45)	1.08, (0.81, 1.44)	1.26 (0.92, 1.72)	1.24 (0.93, 1.66)
Urbanicity: rural vs urban	1.16, (0.85, 1.58)	1.16, (0.85, 1.57)	0.81 (0.69, 0.95)	0.79 (0.67, 0.93)
Income: higher vs lower	1.56, (1.19, 2.06)	1.61, (1.27, 2.03)	1.82 (1.41, 2.35)	1.81 (1.40, 2.35)
Age				
18–34 years	1 (ref)	1 (ref)	1 (ref)	1 (ref)
35–54 years	0.88, (0.75, 1.03)	0.86, (0.75, 0.99)	1.12 (0.98, 1.28)	1.08 (0.95, 1.24)
≥55 years	0.85, (0.71, 1.03)	0.86, (0.74, 1.00)	1.43 (1.06, 1.94)	1.37 (0.92, 2.05)
Work/school behavior in past week				
Did not go to work/school outside	1 (ref)	1 (ref)	1 (ref)	1 (ref)
Went 1–3 days a week	1.49, (1.23, 1.80)	1.31, (1.07, 1.59)	1.35 (1.11, 1.64)	1.21 (1.03, 1.41)
Went 4–7 days a week	1.38, (1.20, 1.59)	1.23, (1.11, 1.36)	1.44 (0.98, 2.11)	1.33 (0.91, 1.96)
Grocery store behavior in past week				
Did not go to work/school outside	1 (ref)	1 (ref)	1 (ref)	1 (ref)
Went 1–3 days a week	1.71, (1.13, 2.59)	1.63, (1.11, 2.40)	1.28 (1.00, 1.66)	1.21 (0.93, 1.57)
Went 4–7 days a week	1.96, (1.12, 3.43)	1.80, (1.07, 3.04)	1.06 (0.88, 1.27)	0.98 (0.79, 1.22)
Vaccine profile experiment				
95% vs 50% effective vaccine	2.70, (2.22, 3.28)	2.68, (2.24, 3.20)	--	--
20% vs 5% risk of side effects	0.95, (0.82, 1.10)	0.95, (0.82, 1.11)	--	--
Country				
U.S.	1 (ref)	1 (ref)	1 (ref)	1 (ref)
Mainland China	4.04, (3.71, 4.40)	5.20, (4.95, 5.47)	7.06 (6.10, 8.17)	7.43 (6.32, 8.73)
Taiwan	0.92, (0.77, 1.11)	1.20, (1.03, 1.39)	0.40 (0.35, 0.45)	0.42 (0.37, 0.47)
Malaysia	1.62, (1.54, 1.71)	1.97, (1.85, 2.09)	1.71 (1.60, 1.83)	1.65 (1.51, 1.82)
Indonesia	3.22, (2.83, 3.67)	3.86, (3.49, 4.27)	2.05 (1.67, 2.51)	2.06 (1.66, 2.56)
India	4.48, (3.92, 5.12)	4.21, (3.84, 4.62)	4.41 (3.79, 5.14)	3.78 (3.23, 4.42)

Notes:

OR, odds ratio; CI, confidence interval.

### Supplemental analysis including state-level incidence of disease

Within the sample from the US (n = 7035), we examined the relationship between the log-transformed 7-day rolling average of cases within a state, and the perceived risk of COVID-19 among residents within that state. S1 Fig shows the relationship across time in the full sample, using local estimated scatterplot smoothing. We subsequently stratified the analysis by political affiliation (S2 Fig) and wave of data collection (S3 Fig).

These figures show little consistent relationship between incidence of cases and susceptibility to infection on a state level. Multivariable regression models also showed no relationship between incidence and vaccination status (S2 Table). In models of just the US, which adjusted for wave, gender, urbanicity, income, political affiliation and race/ethnicity, reported incidence of COVID-19, and work/school and grocery store behaviors, perceived susceptibility mediated 21% (95% CI: 13%-29%, P<0.0001) of the relationship between media exposure and intent to vaccinate, and 7% (95% CI: 3%-11%, P = 0.0002) of the relationship between media exposure and vaccination status in 2021.

## Discussion

Our study shows that COVID-19 vaccine acceptance relates to experiences ranging from individual-level experiences to exposures to COVID-19 cases in the media. Specifically, both the proximity and severity of infections that people underwent or witnessed during the pandemic were related to their vaccine decision-making. However, the associations between personal experiences with COVID-19 and intention to vaccination varied by country. Moreover, it appears that perceived susceptibility to COVID-19 could be a critical mediator of this pathway though future studies are needed to better understand to what extent the perceived susceptibility affects the relationship between personal experience and intention to COVID-19 vaccination.

### Country-specific differences in COVID-19 experiences and vaccination

We note several differences by countries in their exposures not only to COVID-19, but to lockdown measures. A larger proportion of our US sample, for instance, stayed away from work than in the other countries. This could be due to variation in when lockdowns occurred relative to our survey distribution. Notably, compared to other Organization for Economic Cooperation and Development (OECD) countries, a larger proportion of the US workforce transitioned to working from home during the first wave of COVID-19 [24], although there also was substantial variation in lockdown measures by US state [25]. Future research could examine if this shift in the workforce also influenced risk perceptions.

The strongest relationship between experiences and risk perceptions was found in the US compared to other countries. The wide variations in country-specific estimates of the association between personal experiences with COVID-19 and intention to vaccination may reflect the different epidemic histories in each country or how they consume and trust media. The timing of epidemic peaks and the severity of the epidemic likely contribute to country-level differences. Whereas China experienced its harshest epidemic events early on in early 2020, the other countries experienced those most severe points in the epidemic in late 2020 or spring and summer of 2021 [26].

The role of the media presents an interesting vantage point from which to investigate intention to vaccination. In a previous study conducted in Taiwan, receiving pandemic information from the internet, television, or from personal networks was associated with higher self-reported worry compared to information derived from academic resources [27]. Given the low COVID-19 confirmed cases in Taiwan, especially during 2020, media seemed to play an important role in familiarizing individuals with COVID-19 and impacting their vaccine decision-making.

During the first wave of data collection in India (August 2020), India had a large outbreak of COVID-19 that peaked in mid-September. Our study showed high vaccine acceptance, in line with other studies (e.g., ~90% vaccine acceptance according to Goruntla et al. [28]). In our study, individuals from India with a personal COVID-19 diagnosis had significantly higher vaccination intent, and higher, though not significant, risk perceptions. The study by Goruntla et al. confirmed this finding showing that higher perceived susceptibility to infection and severity of COVID-19 infection were important factors associated with vaccine acceptance [28].

### Media and risk perceptions

Our study acknowledges the role media can play in reinforcing risk perceptions and health behaviors. Our findings suggest that media stories that highlight severe cases of disease could be associated with vaccination behaviors. Other research has highlighted other roles that the media can play. One group analyzing the role of social media “infodemics,” defined as COVID-19 rumors, stigma, and conspiracy theories circulating through the Internet, found that among other nations, both India and Indonesia had rather notable levels of detected misinformation on social media, primarily in the form of false or misleading rumors related to themes such as causes of the pandemic, course of illness, and forms of treatment [29]. Media can also contribute positively to health behaviors, especially if it is paired with ready availability of health services, and if it is funded for a longer period [30]. Past research has identified that the deliverer of information in media and how the message is framed are both important for the effectiveness of any media story in promoting healthy behaviors [31]. Our study contributes to this literature by emphasizing the importance of media portraying real persons affected by COVID-19 illness. Additionally, through understanding the important mediating role of risk perceptions, media could incorporate stories of infection into this visual imagery. Although media can play an important role in affecting individuals’ perceptions of vaccines, other factors–including conspiracy beliefs, political affiliation, and receipt of prior vaccines such as for influenza [32].

Examples from other vaccine-preventable diseases may elucidate the mechanisms behind the impact of personal experiences and risk perceptions on intention to vaccinate. For example, a study of H1N1 vaccination among adults in China found that concerns were significantly related to vaccine uptake among those without chronic health conditions, but not among those with chronic health conditions [33]. Instead, worries about health care costs and frequency of health checkups–measures of health care access and affordability–were more strongly related to vaccine uptake among those with high-risk health conditions [33], indicating the importance of considering structural disparities when measuring vaccine coverage. A study in Malaysia found both perceived susceptibility and cues to action as significant predictors of willingness to pay for a hepatitis B vaccine [34]. That study highlights the importance of activation cues in receiving a vaccine, beyond whether someone is aware of or accepting of a vaccine [35]. These activation cues could also be present in the COVID-19 vaccination roll-out campaigns, but we did not separate out these campaigns from other media exposures in our study.

### Strengths and limitations

This study has a number of strengths. We were able to tease apart *tangible* experiences from *perceived* risk of COVID-19. This study draws from a breadth of locations. As vaccine rollout expands worldwide and as literature grows on public attitudes towards COVID-19 vaccines, narratives from an array of cultural contexts are crucial, especially given inequitable vaccine distribution patterns and different epidemic patterns.

One crucial drawback to these findings are imprecise estimates that result from low prevalence of personal and within network COVID-19 infections across the six countries. Additionally, the data for this study are derived from conveniently sampled, internet-based surveys. Individuals without internet access were not able to participate in our study even though the internet coverage and smartphone penetration is relatively high in middle-income Asian countries [36–38]. However, future research is needed to better understand vaccine hesitancy issues among those who do not have internet access. We also note heterogeneity in the pandemic response throughout the world, and especially throughout diverse parts of Africa [39], which were not included in our study. Previous research has documented risk perceptions nevertheless are hugely impactful for vaccine rejection across African countries [40]. Moreover, because this is a cross-sectional study, identifying a causal relationship is not possible, though our survey questions did include temporal elements in the wording, incorporating a time component to the research question. We also acknowledge potential differences across participants in interpretation of wording such as “severe” for the experience exposure questions, and this study did not include in depth questions on media usage. Specific exposures, especially for death, could be very important for risk perceptions and vaccine decision-making, but were not assessed in this study.

## Conclusions

This study is a foray into the ways pandemic experiences relate to COVID-19 vaccine acceptance. Across most countries examined, individuals stating that they remember seeing a severe case of COVID-19 in the media were more likely to be vaccinated. This relationship between media exposure and vaccination status was mediated by increases in perceived susceptibility. These findings speak to the need to highlight personal stories within media. Overall, this study contributes to the theory of how media could affect individual behaviors in a diverse, international sample.

## Supporting information

S1 Fig Relationship between log-transformed 7-day rolling average of COVID-19 cases and perceived susceptibility to infection (n = 7035). (TIF)

S2 Fig Relationship between log-transformed 7-day rolling average of COVID-19 cases and perceived susceptibility to infection, stratified by political affiliation. (TIF)

S3 Fig Relationship between log-transformed 7-day rolling average of COVID-19 cases and perceived susceptibility to infection, stratified by wave of data collection (2 = June 2020, 3 = August 2020, 4 = October 2020, 5 = November 2020, 6 = February 2021, 7 = March 2021, 8 = April 2021, 9 = June 2021). (TIF)

S1 Table Logistic regression models of COVID-19 vaccination by personal experiences, Feb–Jun 2021. (DOCX)

S2 Table Estimates of total effect and direct effect of media experiences on COVID-19 vaccination intention in two logistic regression models. (DOCX)
