# Supplementary figures and images for: A study of COVID-19 vaccination in the US and Asia: The role of media, personal experiences, and risk perceptions
Source: PLOS Glob Public Health. 2022 Jul 13;2(7):e0000734. doi: 10.1371/journal.pgph.0000734 (PMC10021344; doi:10.1371/journal.pgph.0000734)

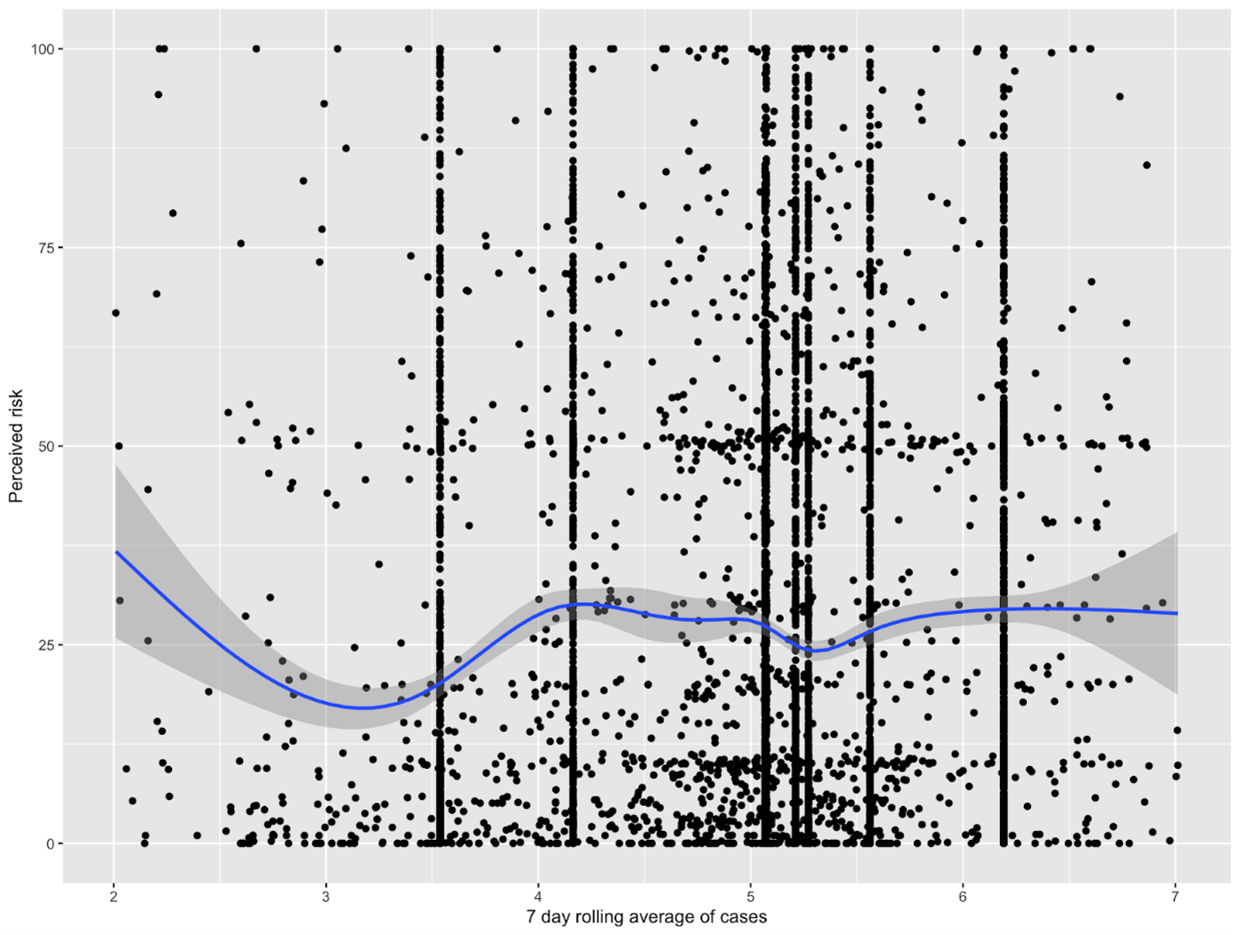

Supplement: S1 Fig — (TIF) [file pgph.0000734.s001.tif]

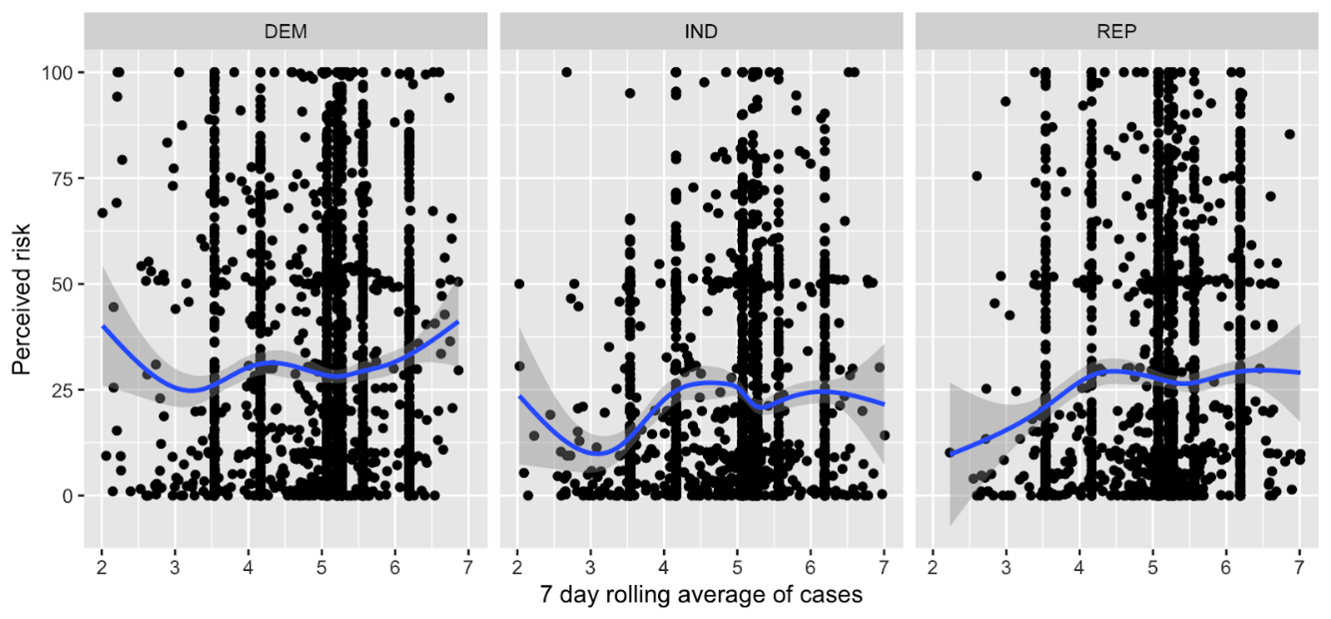

Supplement: S2 Fig — (TIF) [file pgph.0000734.s002.tif]

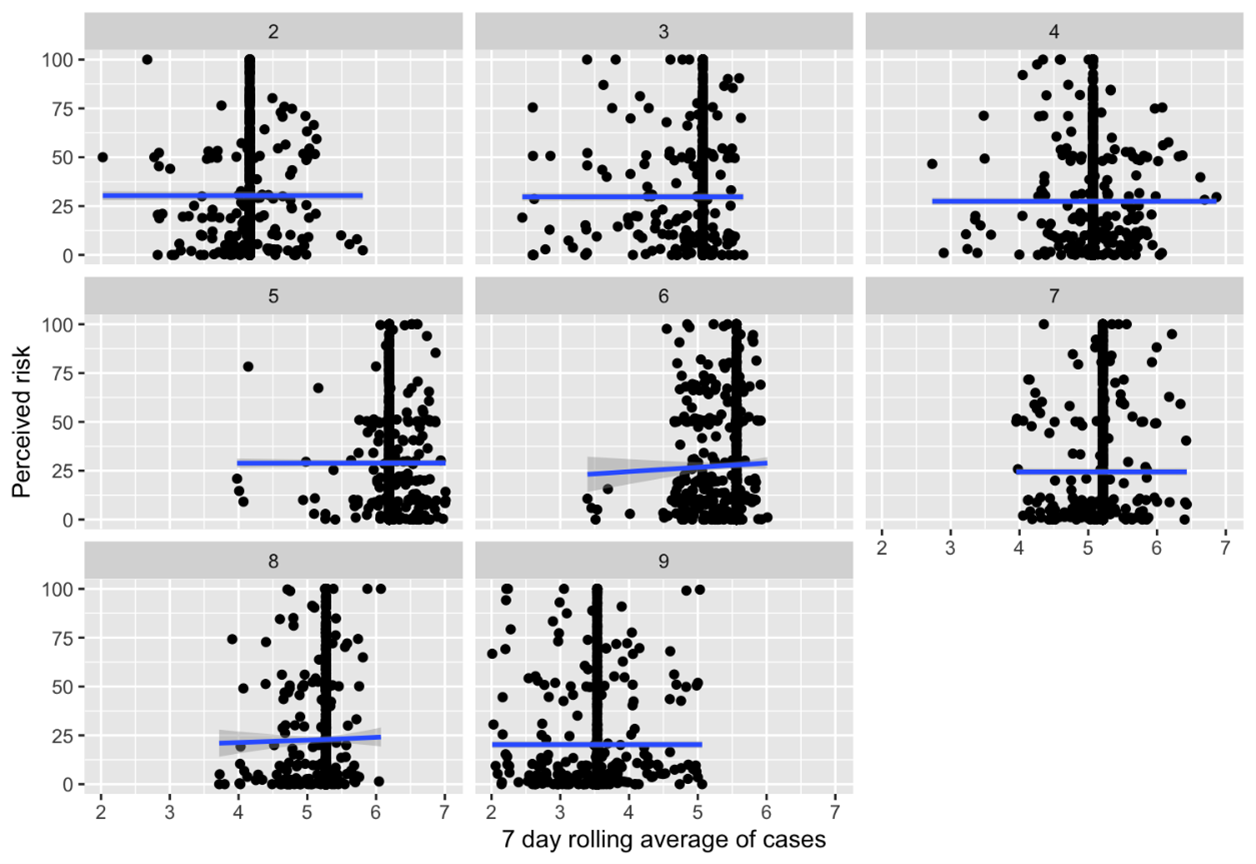

Supplement: S3 Fig — (TIF) [file pgph.0000734.s003.tif]
