# Supplementary material for: A study of COVID-19 vaccination in the US and Asia: The role of media, personal experiences, and risk perceptions
Source: PLOS Glob Public Health. 2022 Jul 13;2(7):e0000734. doi: 10.1371/journal.pgph.0000734 (PMC10021344; doi:10.1371/journal.pgph.0000734)
Supplement: S1 Table — (DOCX) [file pgph.0000734.s004.docx]

**S1 Table.** Logistic regression models of COVID-19 vaccination by personal experiences, Feb – Jun 2021.

|  | U.S.  OR (95% CI) | Mainland China  OR (95% CI) | Taiwan  OR (95% CI) | Malaysia  OR (95% CI) | Indonesia  OR (95% CI) | India  OR (95% CI) |
| --- | --- | --- | --- | --- | --- | --- |
| Sample size | 3665 | 1692 | 1370 | 1528 | 1572 | 1820 |
| Diagnosed with COVID-19 |  |  |  |  |  |  |
| No | 1 (ref) | 1 (ref) | 1 (ref) | 1 (ref) | 1 (ref) | 1 (ref) |
| Yes | 1.44 (1.09, 1.90) | 0.13 (0.02, 0.91) | 2.00 (0.72, 5.59) | 0.70 (0.38, 1.28) | 0.76 (0.43, 1.32) | 2.79 (1.58, 4.93) |
| Friends/family diagnosed with COVID-19 |  |  |  |  |  |  |
| No | 1 (ref) | 1 (ref) | 1 (ref) | 1 (ref) | 1 (ref) | 1 (ref) |
| Yes | 1.00 (0.82, 1.22) | 4.28 (0.41, 44.26) | 1.12 (0.41, 3.01) | 1.72 (1.16, 2.56) | 1.73 (0.99, 3.01) | 1.52 (0.94, 2.44) |
| Seen case of COVID-19 in the media |  |  |  |  |  |  |
| No | 1 (ref) | 1 (ref) | 1 (ref) | 1 (ref) | 1 (ref) | 1 (ref) |
| Yes, and was not severe | 1.23 (0.99, 1.54) | 2.65 (1.26, 5.58) | 1.15 (0.82, 1.62) | 1.58 (0.96, 2.61) | 3.80 (1.81, 7.99) | 1.62 (0.91, 2.87) |
| Yes, and was very severe | 2.87 (2.26, 3.66) | 4.09 (1.61, 10.38) | 1.86 (1.21, 2.85) | 2.38 (1.45, 3.90) | 6.71 (3.03, 14.84) | 2.64 (1.50, 4.64) |

Notes:

OR, odds ratio; CI, confidence interval

Models adjusted for wave of data collection, gender, urbanicity, income, age, work/school behaviors, grocery store behaviors, and vaccine safety and effectiveness profile experiment.
