# Supplementary material for: A study of COVID-19 vaccination in the US and Asia: The role of media, personal experiences, and risk perceptions
Source: PLOS Glob Public Health. 2022 Jul 13;2(7):e0000734. doi: 10.1371/journal.pgph.0000734 (PMC10021344; doi:10.1371/journal.pgph.0000734)
Supplement: S2 Table — (DOCX) [file pgph.0000734.s005.docx]

**S2 Table.** Estimates of total effect and direct effect of media experiences on COVID-19 vaccination intention in two logistic regression models.

|  | Intent to vaccinate | | Already or plan to vaccinate | |
| --- | --- | --- | --- | --- |
|  | Adjusted model 1  (N=3,344) | Adjusted model 2  (N=3,344) | Adjusted model 1  (N=3,654) | Adjusted model 2  (N=3,654) |
|  | OR (95% CI) | OR (95% CI) | OR (95% CI) | OR (95% CI) |
| Seen case of COVID-19 in media |  |  |  |  |
| No / yes, and was not severe | 1 (ref) | 1 (ref) | 1 (ref) | 1 (ref) |
| Yes, and was very severe | 1.70 (1.43, 2.03) | 1.56 (1.31, 1.85) | 2.32 (1.90, 2.83) | 2.24 (1.84, 2.74) |
| Perceived susceptibility (continuous, %) | -- | 1.01 (1.01, 1.02) | -- | 1.01 (1.00, 1.01) |
| Wave |  |  | -- | -- |
| June 2020 | 1 (ref) | 1 (ref) | -- | -- |
| August 2020 | 0.75 (0.54, 1.05) | 0.75 (0.54, 1.04) | -- | -- |
| October 2020 | 0.42 (0.31, 0.59) | 0.44 (0.31, 0.61) | -- | -- |
| November 2020 | 0.40 (0.23, 0.69) | 0.41 (0.24, 0.72) | -- | -- |
| February 2021 | -- | -- | 1 (ref) | 1 (ref) |
| March 2021 | -- | -- | 1.16 (0.89, 1.50) | 1.20 (0.92, 1.56) |
| April 2021 | -- | -- | 1.37 (1.05, 1.77) | 1.42 (1.09, 1.85) |
| June 2021 | -- | -- | 1.97 (1.03, 3.78) | 2.04 (1.06, 3.93) |
| Gender: male vs female | 1.72 (1.45, 2.04) | 1.71 (1.44, 2.04) | 1.93 (1.60, 2.32) | 1.90 (1.58, 2.29) |
| Urbanicity: rural vs urban | 1.08 (0.89, 1.30) | 1.02 (0.84, 1.23) | 0.87 (0.72, 1.05) | 0.83 (0.68, 1.01) |
| Income: higher vs lower | 1.61 (1.30, 1.98) | 1.68 (1.36, 2.07) | 2.33 (1.90, 2.86) | 2.32 (1.89, 2.85) |
| Age |  |  |  |  |
| 18-34 years | 1 (ref) | 1 (ref) | 1 (ref) | 1 (ref) |
| 35-54 years | 0.79 (0.63, 0.99) | 0.77 (0.61, 0.97) | 1.04 (0.83, 1.30) | 1.04 (0.83, 1.30) |
| ≥55 years | 0.83 (0.65, 1.05) | 0.88 (0.69, 1.13) | 2.25 (1.76, 2.86) | 2.38 (1.86, 3.03) |
| Political affiliation |  |  |  |  |
| Democrat | 1 (ref) | 1 (ref) | 1 (ref) | 1 (ref) |
| Independent | 0.59 (0.48, 0.73) | 0.60 (0.49, 0.75) | 0.32 (0.26, 0.41) | 0.34 (0.27, 0.43) |
| Republication | 0.65 (0.52, 0.81) | 0.64 (0.51, 0.80) | 0.34 (0.27, 0.43) | 0.34 (0.27, 0.43) |
| Race / ethnicity |  |  |  |  |
| Hispanic | 1 (ref) | 1 (ref) | 1 (ref) | 1 (ref) |
| Non-Hispanic Asian | 0.75 (0.49, 1.17) | 0.77 (0.49, 1.19) | 2.98 (1.62, 5.49) | 3.00 (1.63, 5.50) |
| Non-Hispanic Black | 0.37 (0.25, 0.55) | 0.39 (0.27, 0.58) | 0.41 (0.27, 0.60) | 0.41 (0.27, 0.60) |
| Non-Hispanic white | 0.79 (0.57, 1.08) | 0.84 (0.61, 1.15) | 0.98 (0.71, 1.34) | 1.00 (0.73, 1.38) |
| Other | 0.28 (0.15, 0.54) | 0.33 (0.18, 0.62) | 0.55 (0.29, 1.04) | 0.57 (0.30, 1.08) |
| Vaccine profile experiment |  |  |  |  |
| 95% vs 50% effective vaccine | 2.44 (2.05, 2.90) | 2.49 (2.09, 2.97) | -- | -- |
| 20% vs 5% risk of side effects | 0.85 (0.72, 1.01) | 0.85 (0.72, 1.01) | -- | -- |
| Log-transformed 7-day rolling average of cases (continuous) | 1.22 (0.95, 1.56) | 1.20 (0.94, 1.53) | 1.01 (0.74, 1.37) | 0.99 (0.72, 1.34) |
| Work/school behavior in past week |  |  |  |  |
| Did not go to work/school outside | 1 (ref) | 1 (ref) | 1 (ref) | 1 (ref) |
| Went 1-3 days a week | 1.50 (1.15, 1.96) | 1.36 (1.04, 1.79) | 1.52 (1.14, 2.02) | 1.41 (1.06, 1.88) |
| Went 4-7 days a week | 1.37 (1.10, 1.71) | 1.31 (1.05, 1.63) | 0.86 (0.69, 1.07) | 0.83 (0.66, 1.03) |
| Grocery store behavior in past week |  |  |  |  |
| Did not go to work/school outside | 1 (ref) | 1 (ref) | 1 (ref) | 1 (ref) |
| Went 1-3 days a week | 1.06 (0.80, 1.42) | 1.06 (0.80, 1.41) | 1.21 (0.89, 1.66) | 1.24 (0.90, 1.70) |
| Went 4-7 days a week | 1.11 (0.78, 1.58) | 1.01 (0.71, 1.43) | 1.25 (0.87, 1.80) | 1.24 (0.86, 1.78) |

Notes:

OR, odds ratio; CI, confidence interval
